# Vaccine Adjuvants Induce Formation of Intraperitoneal Extracellular Traps in Flounder (*Paralichthys olivaceus*)

**DOI:** 10.3389/fcimb.2022.875409

**Published:** 2022-03-30

**Authors:** Qian Li, Heng Chi, Xueyan Shi, Qiujie Gan, Roy Ambli Dalmo, Yuan-yuan Sun, Xiaoqian Tang, Jing Xing, Xiuzhen Sheng, Wenbin Zhan

**Affiliations:** ^1^ Laboratory of Pathology and Immunology of Aquatic Animals, KLMME, Ocean University of China, Qingdao, China; ^2^ Laboratory for Marine Fisheries Science and Food Production Processes, Qingdao National Laboratory for Marine Science and Technology, Qingdao, China; ^3^ Norwegian College of Fishery Science, Faculty of Biosciences, Fisheries and Economics, University of Tromsø, The Arctic University of Norway, Tromsø, Norway; ^4^ Key Laboratory of Experimental Marine Biology, Institute of Oceanology, Chinese Academy of Sciences, Qingdao, China

**Keywords:** extracellular traps (ETs), antigen, adjuvant, vaccination, fish

## Abstract

Adjuvants are used to increase the strength, quality, and duration of the immune response of vaccines. Neutrophils are the first immune cells that arrive at the injection site and can release DNA fibers together with granular proteins, so-called neutrophil extracellular traps (NETs), to entrap microbes in a sticky matrix of extracellular chromatin and microbicidal agents. Similar extracellular structures were also released by macrophages, mast cells, and eosinophils and are now generalized as “ETs.” Here we demonstrated that Alum adjuvant stimulation led to peritoneal cells swarming and ET release *in vitro*. Moreover, compared to antigen stimulation alone, ET release was significantly increased after stimulation with antigen-mixed adjuvants and in a time- and dose-dependent manner. *In vivo*, we were able to monitor and quantify the continuous changes of the ET release in the same fish by using the small animal *in vivo* imaging instrument at different times during the early stages after intraperitoneal immunization. The results showed that the fluorescence signal of ETs in the peritoneum increased from 0 to 12 h after injection and then gradually decreased. The fluorescence signals came from extracellular DNA fibers, which are sensitive to DNase I and confirmed by microscopy of peritoneal fluid *ex vivo*. In summary, this study introduced a new method for detecting ETs in the peritoneum of fish *in vivo* and indicated that ET formation is involved in the immune response at the early stage after intraperitoneal immunization to vaccines.

## Introduction

Vaccination is one of the most adequate methods to control infectious diseases that threaten the fish aquaculture industry worldwide. Since pure antigens alone are not sufficient for protection, adjuvants or immunostimulants are needed to increase vaccine potency and efficacy (Schijns, 2001). Injection of vaccine into the intraperitoneal (i.p.) cavity of fish results in rapid changes of the cellular composition in the peritoneum and an increase in the number of neutrophils, lymphocytes, macrophages, and other immune cells often accompanies robust immune response at both humoral and cellular levels (Meseguer et al., 1993; Afonso et al., 1998; Thorarinsson and Powell, 2006; Tumbol et al., 2009). Oil-adjuvanted vaccines are routinely used for most commercial vaccines available for the salmonids farming industry (Stils, 2005; Mutoloki et al., 2006; Brudeseth et al., 2013; Noia et al., 2014). Aluminum salts (Alum) adjuvants consist of microaggregates, and upon administration of a vaccine, the aggregates containing antigens may be phagocytosed by sentinel cells such as macrophages or neutrophils. The Alum formulation will persist intraperitoneally over time. As the pivotal cells of the innate immune system, macrophages and neutrophils link the innate and adaptive immune systems, induce inflammation, and activate the antigen-presenting signal, thus mediating the initiation of the adaptive immune system (Mutoloki et al., 2004; Thorarinsson and Powell, 2006; Tafalla et al., 2013; Thim et al., 2014). Although Alum has been extensively used in human vaccination for decades, the use of Alum in commercial fish vaccines is not commonly used. It has been reported that Alum adjuvants produce fewer internal side effects, such as the formation of granulomas, compared to the classical Freund’s adjuvant as reported for turbot *Scophthalmus maximus* (Angosto et al., 2018).

Neutrophils are the first immune cells to arrive at a site of infection and constitute an essential part of the innate immune system with their capability of destroying infectious threats through, e.g., phagocytosis, degranulation of cytotoxic substances, and activity of reactive oxygen species (ROS) (Nathan, 2006; Borregaard, 2010; Arazna et al., 2013; Kolaczkowska and Kubes, 2013; Pijanowski et al., 2013). In addition, neutrophils may release extracellular chromatin fibers with elastase, cathepsin G, lactoferrin, gelatinase, and myeloperoxidase (MPO) from neutrophilic granules neutrophil extracellular traps (NETs) after infection by pathogens, parasites, and viruses (Brinkmann et al., 2004; Galluzzi et al., 2018). Chemokine CXCb1, lipopolysaccharide, and interferon-γ2 also stimulate the formation of NETs in neutrophils (Pijanowski et al., 2015; Pijanowski et al., 2020). These gladiators of innate immunity can throw catching and poisonous NETs and produce components of the humoral arm, which may modulate T-cell responses (Leliefeld et al., 2015). However, if dysregulated, overexuberant neutrophil recruitment contributes to collateral tissue damage, defective healing, chronic inflammation, and vasculature injury (Kolaczkowska et al., 2015; Peiseler and Kubes, 2019). Recently, studies have demonstrated that also mast cells, eosinophils, and macrophages can produce similar extracellular traps (ETs) in response to pathogens or proinflammatory stimuli (Von Köckritz-Blickwede et al., 2008; Yousefi et al., 2008; Loureiro et al., 2019).

ETs have been reported in several teleost species including turbot, tongue sole, flounder, fathead minnows, carp, and zebrafish in the past decade. However, our understanding of ET formation in fish remains with their antimicrobial activity (Palic et al., 2007a; Palic et al., 2007b; Jiao et al., 2010; Brogden et al., 2012; Pijanowski et al., 2013; Chi and Sun, 2016; Zhao et al., 2017; Hao et al., 2021). The overall role of ETs in host defense remains a topic of debate since the actual cause of their formation still is unclear. An important question, which presently is far from being answered, is: How does an early innate immune response of ET formation to the model vaccine containing adjuvants show off? In the present study, we aimed to establish a simple and accurate method for detecting and assessing early release characteristics of ETs following vaccination. As such, we examined and tracked ET formation in peritoneum using *in vivo* imaging after i.p. administration of ovalbumin (OVA) antigen with mineral oil and Alum adjuvants in flounder (*Paralichthys olivaceus*). In addition, qualitative and quantitative analyses of ETs released from peritoneal cells *in vivo* and *ex vivo* were studied.

## Material and Methods

### Fish

Healthy flounders of body length 5–6 cm or 30–32 cm were purchased from a fish farm in Qingdao, Shandong Province, China. The flounders were acclimated in aerated seawater at 22°C for 1 week and used for the subsequent studies. Fish were anesthetized with tricaine methanesulfonate (Sigma, St. Louis, MO, USA) prior to experiments involving injection and then were sacrificed. The animal experiments were approved by the Instructional Animal Care and Use Committee of the Ocean University of China. All possible endeavors were made to minimize suffering and maintain animal welfare.

### Antigen, Adjuvant, and Mixture Formulation

The preparation of aluminum hydroxide was done following the protocol reported previously (Reithofer et al., 2020). In brief, 5% NaOH and 5% Al_2_(SO_4_)_3_ were sterilized by passing through a 0.22-μm filter. After being heated at 60°C for 30 min, two volumes of 5% NaOH and five volumes of 5% Al_2_(SO_4_)_3_ were mixed with stirring, followed by centrifugation at 10,000 *g* for 5 min. After being washed twice with sterile phosphate-buffered saline (PBS), the mixture was suspended in L-15 medium (HyClone, Logan, UT, USA) to give 8 mg ml^−1^. For fluorescence-dyed Alum formulation, a total of 10 mg of fluorescein isothiocyanate (FITC) (MP Biomedicals, Solon, OH, USA) was added to Alum suspension and incubated for 24 h in the dark at room temperature on a rotator. Free FITC was removed from FITC-Alum by 12,000 *g* of centrifugation for 10 min and then washed 10 times or until no fluorescence was present in the supernatant. The pellets were resuspended in PBS and used to stimulate peritoneal cells. For OVA/Alum formulation, Alum was mixed with an equal volume of 10 mg ml^−1^ of OVA (grade V; Sigma, Poole, UK) and incubated at room temperature for 20 min. After centrifugation at 14,000 *g* for 10 min, sedimented OVA/Alum was resuspended in PBS. For OVA/oil formulation, OVA was emulsified with Freund’s incomplete adjuvant (FIA; Sigma, St. Louis, MO, USA) at equal volume.

### Isolation of Peritoneal Cells

Flounders of 30–32 cm in length were intraperitoneally injected with 40 ml of L-15, and the abdomen was gently massaged to get peritoneal cells exuded. Peritoneal fluid was withdrawn using a syringe and thereafter centrifuged at 480 g for 10 min. The cell pellets were resuspended in 5 ml of L-15 medium and washed again by centrifugation at 480 g for 5 min. The purified peritoneal cells were resuspended in 5 ml of L-15 medium before seeding.

### Immunofluorescence

Since ETs were fragile, each step was done with maximal care to preserve the nanostructures. Peritoneal cells (1.5 × 10^6^) were seeded onto round glass coverslips (12 mm) that previously had been treated with 0.001% poly-l-lysine (Sigma, St. Louis, MO, USA) in 24-well cell culture plates (Corning Costar, Cambridge, MA, USA). After the cells had been attached, the peritoneal cells were stimulated with 250 nM ml^−1^ of phorbol myristate acetate (PMA) (Sigma, St. Louis, MO, USA), 10 mg ml^−1^ of OVA, 10 mg ml^−1^ of Alum, or 10 mg ml^−1^ of OVA/Alum for 3 h. Non-stimulated cells served as controls. For fluorescence microscopy, the peritoneal cells were fixed with 4% paraformaldehyde (Sigma, St. Louis, MO, USA). The following steps were performed in a humid chamber at room temperature. Washing of coverslips was performed three times by pH 7.4 PBS for 5 min, and then unspecific binding was blocked with 5% bovine serum albumin (BSA) for 60 min. The coverslips were incubated for 1 h with 1:500 mouse monoclonal antibody against histone 3 (H3) (EASYBIO, Beijing, China). After being washed three times, the coverslips were incubated for 1 h with 1:1,000 anti-rabbit IgG labeled by Alexa Fluor 649 (Sigma, St. Louis, MO, USA). DNA was stained by 4′,6-diamidino-2-phenylindole (DAPI; BioLegend, Santiago, Chile) for 5 min. After DAPI staining, the slides were observed by fluorescence microscopy (Zeiss, Jena, Germany).

### Electron Microscopy

The peritoneal cells were seeded at a density of 1.5 × 10^6^ cell ml^−1^ on glass coverslips and stimulated with 10 mg ml^−1^ of OVA/Alum for 3 h. After treatment, the cells were fixed with 2.5% glutaraldehyde (Hushi, Shanghai, China) in PBS for 2 h and dehydrated in a series of increased concentrations of ethanol (30%, 50%, 70%, 80%, 90%, and 100%) for 10 min at 4°C in each step. The cells were treated with isoamyl acetate for 10 min, critical point-dried (Hitachi-HCP, Hitachi, Tokyo, Japan), sputter-coated with platinum (MC1000, Hitachi, Tokyo, Japan), and examined with a scanning electron microscopy (SEM) (S-3400N, Hitachi, Tokyo, Japan).

For the transmission electron microscopy (TEM) study, the peritoneal cells were stimulated with 10 mg ml^−1^ of OVA/Alum in centrifuge tubes for 3 h. After centrifugation at 480 g for 10 min, the pellets were fixed with 2.5% glutaraldehyde (Hushi, Shanghai, China) in PBS for 2 h and washed three times for 30 min in PBS at 4°C. Then, the cells were post-fixed with 1% osmium tetroxide (Ted Pella, CA, USA) in PBS for 2 h and washed three times with PBS at 4°C. Following this, the samples were dehydrated in alcohol (Hushi, Shanghai, China), infiltrated with acetone (Tieta, Laiyang, China) and epoxy resin (SPI-CHEM, California, USA) mixture, and embedded in polymerizing epoxy resin. Ultrathin sections were obtained by a Leica EM UC7 ultramicrotome (Leica Microsystems, Berlin, Germany) and were transferred onto copper grids covered with the Formvar membrane (Electron Microscopy China, Beijing, China); 2% uranyl acetate and lead citrate (Ted Pella Inc., California, USA) were used for contrast staining. The sections were photographed with a TEM (HT7700, Hitachi, Tokyo, Japan).

### Fluorimetric Assay for the Quantification of Extracellular Traps *In Vitro*

The quantification of ETs was performed as reported previously (Parker et al., 2012). Briefly, peritoneal cells (1 × 10^6^ cells ml^−1^) were suspended in L-15 medium and seeded in a black 96-well plate (200 μl well^−1^) (Cayman Chemical, Ann Arbor, MI, USA). The cells were treated with 500 μg ml^−1^ of OVA, or 500 μg ml^−1^ of OVA/Alum for 1, 2, or 4 h. The untreated cells served as control. For the concentration-dependent study of Alum adjuvant, 25, 50, or 100 μg ml^−1^ of Alum was used to stimulate the cells. After treatment, 10 μl of Sytox Green with 80 mg ml^−1^ was added to each well, followed by incubation for 5 min. Fluorescence was then quantified as relative fluorescence units (RFU) at 485-nm excitation and 530-nm emission using a fluorescence spectrophotometer (POLARstar OPTIMA, Ortenberg, Germany).

### *In Vivo* Imaging and Relative Quantification of Extracellular Traps

Flounders of 3–5 cm in length were randomly divided into four groups (10 individuals for each group). On the day of immunization, 10 mg ml^−1^ of OVA, 10 mg ml^−1^ of OVA/FIA, and 10 mg ml^−1^ of OVA/Alum or PBS were injected into the fish in each group (50 μl per individual). The dye SYTOX Green was intraperitoneally injected along with the formulations to stain extracellular DNA. Multiphoton imaging was performed on a small animal *in vivo* imaging instrument (Vilber Bio Imaging Fusion FX6, Collegien, France). Whole-body images were obtained at 0, 6, 12, 24, 36, 48, and 72 h after injection-using excitation and emission wavelengths of 488 and 523 nm, respectively. To determine ET degradation by DNaseI, flounders were intraperitoneally injected with 10 μl of PBS medium containing 100 U ml^−1^ of DNase I at 12 h after 10 mg ml^−1^ of OVA/Alum injection. Images were obtained every 1 min for up to 8 min.

### Visualization of Extracellular Traps *Ex Vivo*

Twelve hours after intraperitoneal injection of the formulations, Sytox Green was injected into the peritoneal cavity. After 15 min, the peritoneal fluid was obtained and seeded on glass coverslips. The evaluation of ET formation was done by fluorescence microscopy (Zeiss, Jena, Germany).

### Statistical Analysis

All experiments were performed more than three times, and statistical analyses were performed using the one-way ANOVA followed by least significant difference (LSD) multiple group comparisons in the SPSS 17.0 software (SPSS Inc., Chicago, IL, USA). Data were expressed as mean ± SD, and the difference was considered significant when *p* < 0.05.

## Results

### Alum Induces Immune Cell Swarming

**Figure 1:**
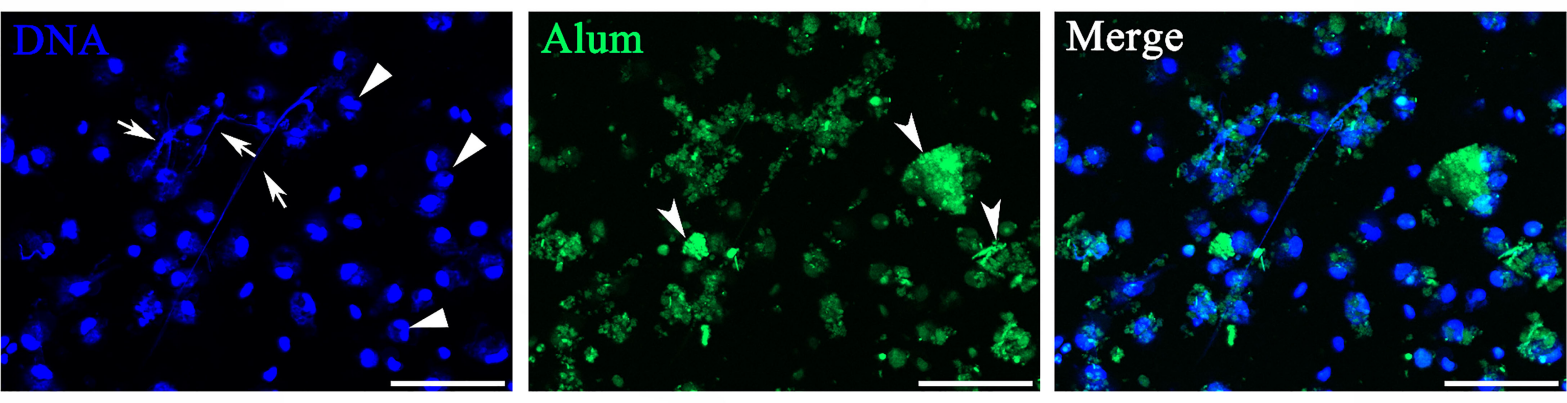
Peritoneal cells stimulated with Alum led to attraction of neutrophils and extracellular trap formation *in vitro*. Fluorescence microscopy of peritoneal cells after stimulation with fluorescently labeled Alum at 3 h illustrating the interaction of Alum particles with extracellular traps (ETs). Arrows with tail indicate ETs, triangles indicate neutrophils, and arrows without tail indicate patches of Alum particles. Scale bars, 20 µm.

**Figure 2:**
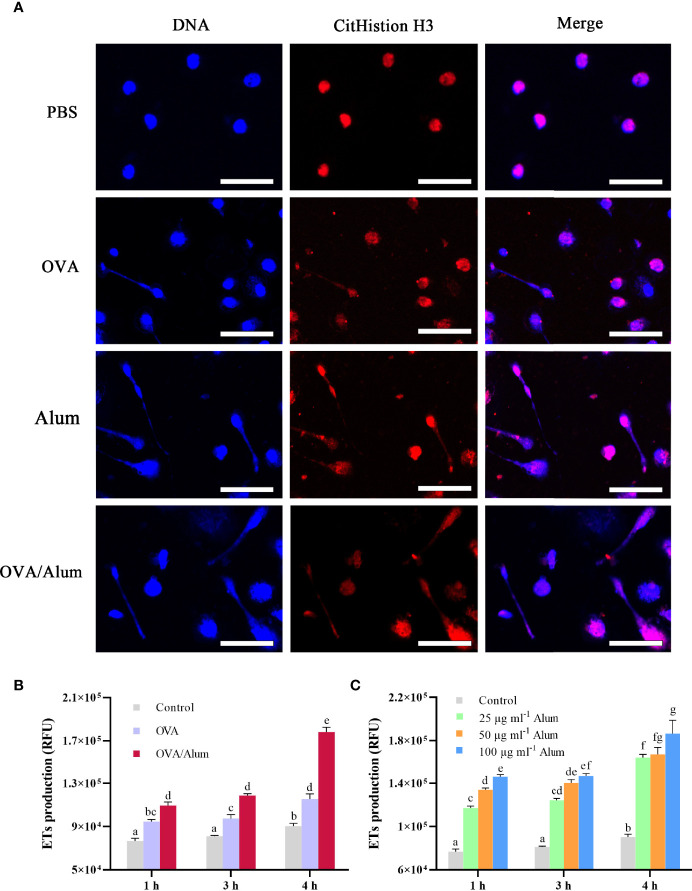
Ovalbumin (OVA) and Alum trigger extracellular trap (ET) formation of peritoneal cells *in vitro*. **(A)** Fluorescence microscopy of peritoneal cells after incubation with OVA, Alum, OVA/Alum mixture, and phosphate-buffered saline (PBS) at 3 h. Co-localization of expelled DNA (blue) with the CitHistion H3 (red) was observed. Scale bars, 20 µm. **(B)** Time course production of ETs by peritoneal cells after stimulation with OVA, Alum, or OVA/Alum for different times. ET production was measured by fluorescence of Sytox Green bound to extracellular DNA in plate reader assays. **(C)** Production of ETs was quantified after stimulation with different concentrations of Alum. The results shown are from three independent experiments. Different letters above the bar represent the statistical significance (*p* < 0.05). Error bars represent standard errors of SD.

Isolated peritoneal cells exposed to FITC-dyed Alum were investigated *in vitro* with special attention to whether they were associated with Alum aggregates. As shown in **Figure 1**, most DAPI-stained cells were identified in or close to colloidal Alum particles at hour 3 after stimulation of FITC-dyed Alum. Some cells exhibited nuclear characteristics as producing ETs, including reniform indented nuclei, the release of fiber-like structures, and cell clustering. The peritoneal cells treated by PBS are shown in **Figure 2A**. Overall, the imaging data demonstrate that Alum rapidly induces recruitment of neutrophils like cells and that Alum induced “neutrophils swarm” cell aggregates in close connection to Alum deposits.

### Extracellular Trap Response to Antigen and Adjuvant From Peritoneal Cells

The formation of ETs was detected by using a fluorometric method at 3 h after stimulation with OVA, Alum, and OVA/Alum *in vitro*. Except for the control cells treated with PBS, extracellular DNA fibers were observed from the wells containing cells in all stimulated groups. DNA from disintegrated nuclei or web-like extracellular DNA fibers labeled by DAPI were co-localized with citrullinated histone H3 (CitH3) labeled by Alexa Fluor^®^ 649 goat anti-mouse IgG (**Figure 2A**).

As shown above, OVA and Alum induced apparent production of ETs in peritoneal cells. The quantitation of ETs in control and stimulated groups was measured by a fluorescence spectrophotometer. The DNA release of OVA- and OVA/Alum-stimulated cells was significantly higher than that of the control cells at all the time points (*p* < 0.05) and increased significantly with time after injection throughout the study period (*p* < 0.05) (**Figure 2B**). In addition, the DNA release in the OVA/Alum-stimulated group was significantly higher compared to that of the OVA-stimulated group at the corresponding time points (*p* < 0.05). In the Alum concentration study (**Figure 2C**), the presence of Alum increased in a dose- and time-dependent manner with the release of ETs. In the Alum-stimulated groups with 25, 50, and 100 μg ml^−1^, ET release increased from hour 1 to hour 3, and a significant difference was observed at hour 4 compared to other time points (*p* < 0.05). The DNA released from 100 μg ml^−1^ of Alum-stimulated cells was significantly higher than that from 25 μg ml^−1^ of Alum-stimulated cells at all the time points (*p* < 0.05). The cells stimulated with 50 μg ml^−1^ of Alum were significantly higher than the cells stimulated with 25 μg ml^−1^ of Alum at hours 1 and 3. Alum-stimulated cells measuring 100 μg ml^−1^ produced significantly more ETs as compared to 50 μg ml^−1^ of Alum-stimulated cells at hour 1.

### Ultrastructural Observation of Extracellular Trap Release From Peritoneal Cells

**Figure 3:**
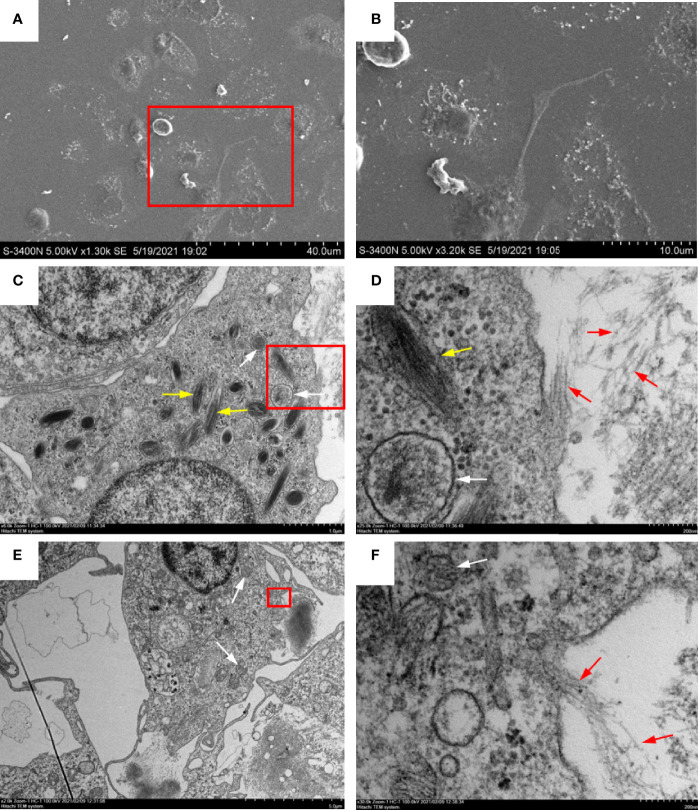
Ultrastructural observation of extracellular trap (ET) release from peritoneal cells in response to ovalbumin (OVA)/Alum stimulation. **(A, B)** Scanning electron microscopy showed that long stretches of fibers were released from peritoneal cells. **(C, D)** Transmission electron microscopy of ETs releasing neutrophil-like cells. **(E, F)** Transmission electron microscopy of ETs releasing macrophage-like cells. **(B**, **D, F)** Magnified images of the boxed regions in **(A**, **C**, **E**) respectively. White arrows indicate mitochondria, yellow arrows indicate fusiform peroxisomes, and red arrows show DNA fibers released from a pore in the cell membrane.

To identify the morphology and types of ETs releasing peritoneal cells stimulated with OVA/Alum, ultrastructural studies using EM were performed. High-resolution SEM showed that flounder peritoneal cells released long stretches of fibers in response to OVA/Alum (**Figures 3A, B**). TEM revealed that such fibers were mainly released from two types of peritoneal cells. One type was 5–10 μm in diameter, with the irregular cell membrane, while the cytoplasm possessed many peroxisomes containing fusiform filaments (**Figures 3C, D**). The other types of cells showed blunt pseudopods on the surface of the plasma membrane and round nucleus with some heterochromatin clumps. The cytoplasm contained many mitochondria, free ribosomes, but no peroxisomes (**Figures 3E, F**). Both types of cells seemed to release fibers through the pores in the cell membrane.

### Adjuvants Enhanced the Release of Extracellular DNA *In Vivo*

**Figure 4:**
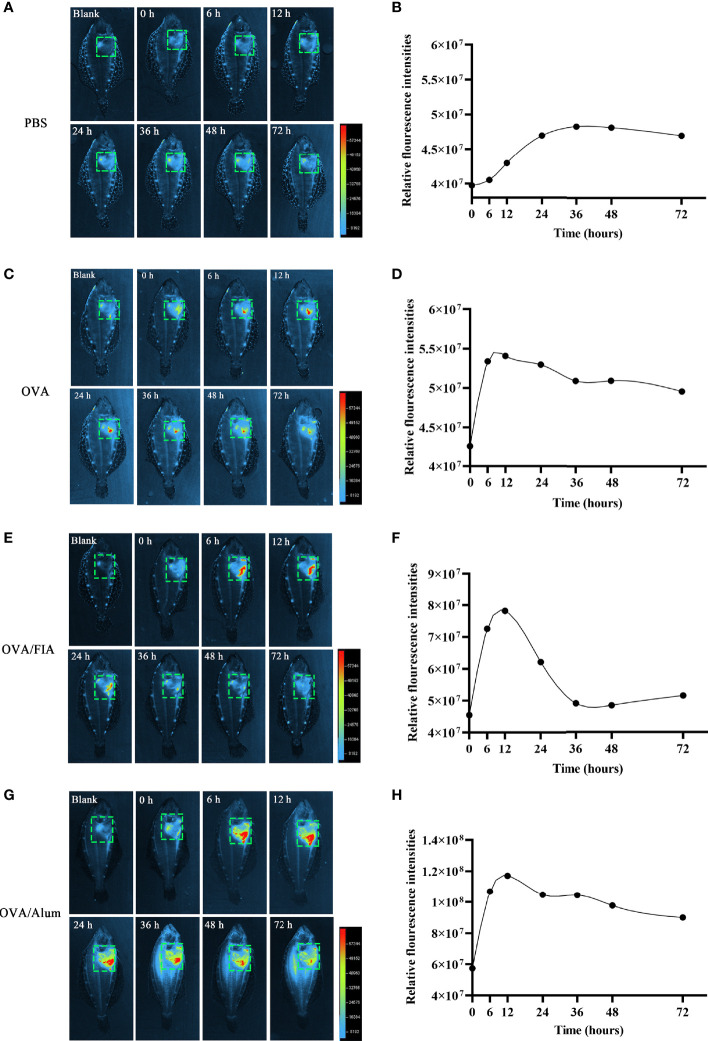
Extracellular trap (ET) release post-intraperitoneal injected with antigen and mixture at different times in flounder. *In vivo* imaging of ETs timely released in flounder after phosphate-buffered saline (PBS) **(A)**, ovalbumin (OVA) **(C)**, OVA/FIA **(E)**, OVA/Alum, and **(G)** intraperitoneal injection. Quantitation of the signals emitted from flounder in the green squares after immunization with PBS **(B)**, OVA **(D)**, OVA/FIA **(F)**, and OVA/Alum **(H)**. The fluorescence intensity indicates the quantity of the extracellular DNA dyed by Sytox Green. The scale represents the number of photons per second/per cm^2^. These representative data are from the study of 10 independent fish.

**Figure 5:**
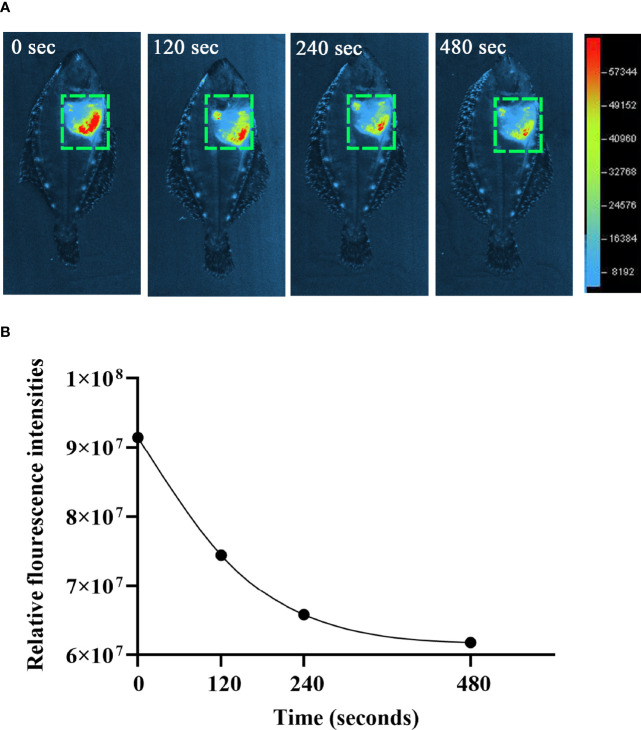
The degradation of extracellular DNA structures at intraperitoneum after exogenous DNaseI injection. **(A)** After ovalbumin (OVA)/Alum-immunized for 12 h, flounder was injected with Sytox Green and DNaseI. The images were taken by small animal *in vivo* imaging instruments at different times. **(B)** Quantitative analysis of fluorescence in flounder peritoneum at different times after DNaseI injection. These representative data are from the study of 10 independent fish.

The observation of ET formation of peritoneal cells *in vitro* prompted us to develop a method to investigate such a process *in vivo*. After OVA, OVA/FIA, and OVA/Alum were injected into the peritoneal cavity, fluorescence signals were detected by using a small animal imaging instrument and quantified from the membrane-impermeable DNA dye (Sytox Green). The fluorescence signal could be detected from hour 6, reached a maximum at hour 12, and then gradually decreased till hour 72 after OVA, OVA/FIA, or OVA/Alum injection (**Figure 4**). The fluorescence from PBS-injected fish was negligible throughout the study period at hour 6 to hour 72 after injection (**Figures 4A, B**). After OVA injection, fluorescence signals due to extracellular DNA were detected in the peritoneum, where the highest fluorescence intensity was 5.4 × 10^7^ photons/s/cm^2^/sr at hour 12 (**Figures 4C, D**). The treatment with OVA/FIA (**Figures 4E, F**) and OVA/Alum (**Figures 4G, H**) induced enhanced extracellular DNA release and the fluorescence intensity reached 7.8 × 10^7^ and 1.1 × 10^8^ photons/s/cm^2^/sr at hour 12, respectively. To verify that fluorometric determination reflected extracellular DNA, a group of fish received DNase I to degrade DNA, which will decrease the fluorescence intensity in the peritoneal cavity. After OVA/Alum-treatment at hour 12, the fish were injected with DNase I intraperitoneally. As shown in **Figures 5A, B**, the fluorescence intensity decreased time-dependently from 9.1 × 10^7^ to 6.2 × 10^7^ photons/s/cm^2^/sr within 480 s, which suggested that the fluorescence originated from extracellular DNA fibers.

### Microscopy of Extracellular Traps *Ex Vivo* From Peritoneum

**Figure 6:**
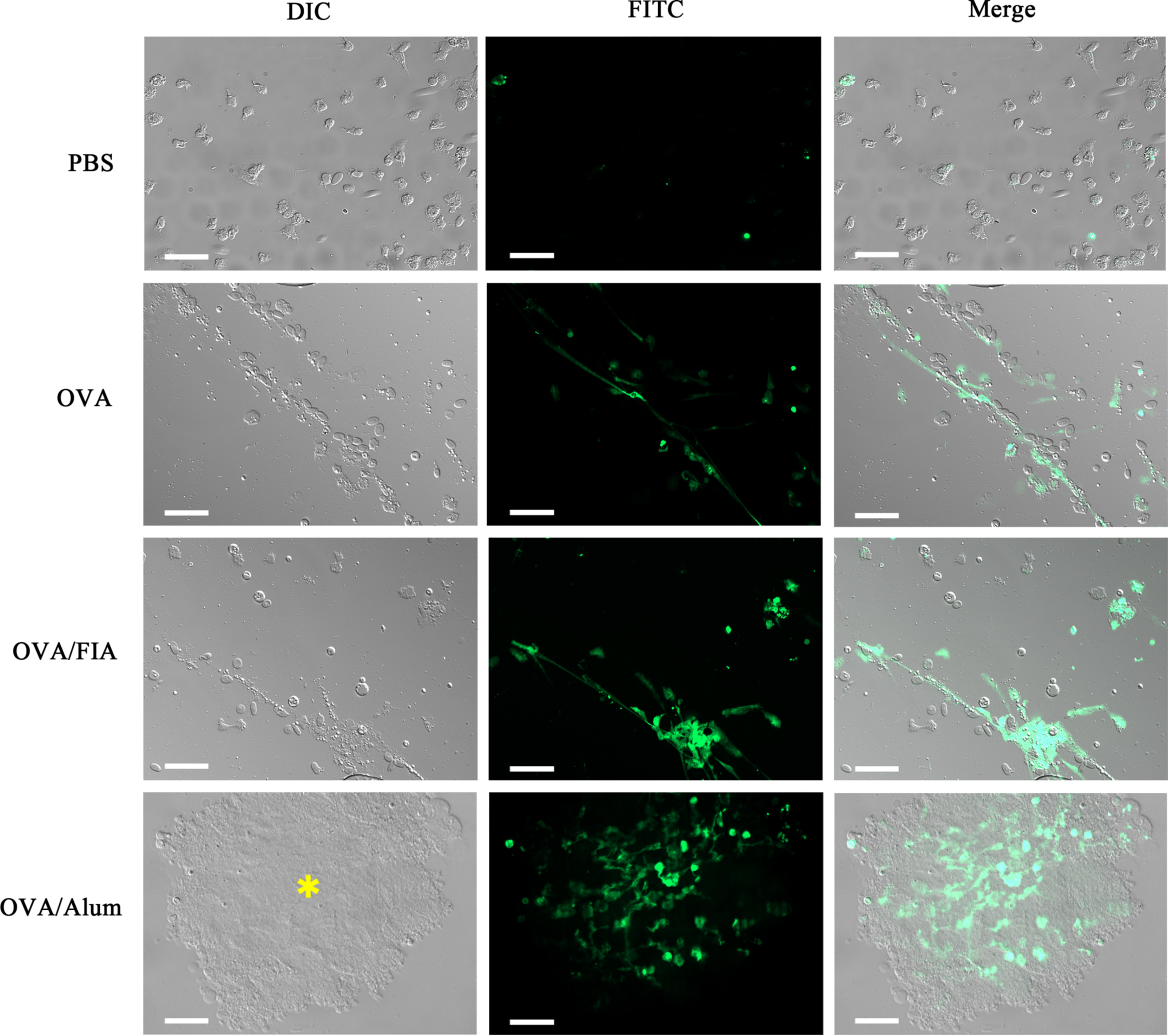
The observation of peritoneal cells and extracellular traps (ETs) *ex vivo* after injection with antigen, adjuvant, or mixture. Representative images of peritoneal cells and ETs isolated from individuals injected with antigen, adjuvant, or mixture after 12 h and dyed with Sytox Green. Arrows indicate the presence of ETs. Asterisk represents Alum and Alum-attracted peritoneal cell mass. Scale bars, 20 µm.

Peritoneal exudates were observed by fluorescence microscopy at hour 12 after immunization to determine the reliability of the mode of detection of ETs *in vivo*. The cells obtained after vaccination with OVA, OVA/FIA, and OVA/Alum possessed typical features of ET formation, such as cell adherence, flattening, and DNA extrusion into the extracellular space (**Figure 6**). The fluorescence intensity was the highest in extracellular DNA fibers, and dead small cells were encountered. The control cells from PBS-injected fish exhibited no DNA fiber, thus showing low fluorescence. We observed, similarly to above, some dead cells that were stained with the fluorescent dye. The released DNA fibers from OVA/Alum-stimulated immune cells were clustered together and located in or close to Alum aggregates, in line with the observation of *in vitro* study shown in **Figure 1**.

## Discussion

A simple small animal imaging method to visualize dye binding to ETs inside the live fish was successfully established. Optical imaging of small molecular fluorescent probes for *in vivo* studies permits a non-invasive assessment of biological processes and may lower the number of animals in experiments compared to *in vitro* studies where multiple time points and dozens of individuals are needed to monitor ET formation (Hassan and Klaunberg, 2004). However, *in vivo* fluorescence imaging is limited by tissue thickness, auto-fluorescence, fluorescence quenching, and light scattering, making visualization of deeply located organs challenging (Bremer et al., 2003). Flounders, especially in adulthood, possess brown skin, thick scales, and black speckles all around the body surface. This may lead to a lower signal-to-noise ratio, which is a known challenge in animal imaging. In this study, we demonstrated that flounder with a length of 5–6 cm, by placing the white ventral surface upward in the small animal *in vivo* imager, was optimal for solving the problem of signal attenuation. Using this method, we were able to monitor and quantify the continuous changes of the ET release in one individual fish at different time points during the early stages after immunization and compared these findings to the fluorometric assay *in vitro*.

OVA has been utilized as a convenient and cost-effective model antigen to evaluate experimental vaccine delivery systems. In some mammalian species, prolonged exposure to OVA may lead to the development of dermatitis and infiltration of CD3^+^ T cells, eosinophils, and neutrophils, as well as increased mRNA levels of interleukin IL-4, IL-5, and IFN-γ (Spergel et al., 1998). OVA peptides may also stimulate phagocytic macrophage and induce the accumulation of neutrophils at the OVA injection site (Mine and D’Silva, 2007). This suggests that OVA elicits both innate and adaptive immune responses. In our study, co-staining with CitH3 of DNA indicated that OVA induced ET formation *in vitro*. Similarly, observations suggested that i.p. injection of OVA also led to ET formation *in vivo* quite early post-injection. Adjuvants are “helpers” whose main purpose is to increase the strength, quality, and duration of the immune response induced by the vaccines (Seubert et al., 2008). This enhancing effect of adjuvants relates especially to a more robust adaptive immune response. In farmed fish, a water-in-oil emulsified vaccine can induce high serum antibody titer against bacterial pathogens (Rahman et al., 2000; Jaafar et al., 2015; Erkinharju et al., 2017; Xu et al., 2019). Certain oil adjuvants may also stimulate the secretion of chemokines such as CCL2, CCL3, CCL4, and CXCL8 (IL-8) by monocyte-derived macrophages in humans, which may precede cellular responses (Seubert et al., 2008; Jiao et al., 2010). Effects of oil adjuvants/oil-adjuvanted vaccines on ET formation have not yet been of major focus. In our study, the fluorescence signal intensity in flounder peritoneum injected with OVA/FIA was significantly higher at all time points compared to OVA alone. This suggests that the FIA contributed to a strengthened ET formation at early time points, indicative of innate response.

Alum adjuvants have been shown to trigger innate immune responses *via* the NLRP3 inflammasome, which results in the recruitment and activation of macrophages and increased expression of, e.g., major histocompatibility complex (MHC) class II (Ko et al., 2017). It has been found that Alum injection induces the production of proinflammatory chemokines and immune cells recruitment (Stephen et al., 2017). Of the immune cells recruited to the inflammatory foci, neutrophils were in the highest numbers 2 and 6 h post-injection of Alum. Macrophages (and other MHC class II cells) arrived after the neutrophils (Lu and HogenEsch, 2013). As such, there is a potential for MHC II antigen presentation and antibody response locally, which is suggested by Erkinharju et al. (2019). Furthermore, Alum has been reported to induce fibrinous ETs, which may encapsulate Alum particles (Munks et al., 2010). The biological impact of an association of fibrinous structures with Alum observed in the present study is not known.

In our study, we found that neutrophil-like cells were the principal cells recruited within 3 h after Alum stimulation, and they released ETs. In addition, both Alum and OVA/Alum mixture induced ETs to release after co-incubation with peritoneal cells for 3 h *in vitro*. Quantitative analysis revealed that ET production was time- and Alum dose-dependent. Moreover, compared to OVA stimulation alone, ET release was significantly increased after stimulation with OVA/Alum. Consistent with the results observed *in vivo*, the fluorescent signal caused by dye-binding to ETs in the peritoneum of the OVA/Alum-injected flounders was significantly stronger than the signal observed in OVA-injected flounders. The fluorescence signal of peritoneal ETs reached a maximum of 12 h post-injection, indicating that ET formation occurred at a very early stage during fish vaccination. Whether the intensity of ET formation may relate to increased antigen presentation, antibody response and efficacy remain to be researched.

Neutrophils are the main cell type that releases ETs, but other granulocytes, as well as mononuclear phagocytes, reportedly share the ability to form ETs and/or to shed chromatin in response to various stimuli (Brinkmann et al., 2004; Von et al., 2008; Yousefi et al., 2008; Schorn et al., 2012). Peritoneal cells comprise a unique mixture of macrophages, monocytes, neutrophils, and lymphocytes (Selvarajan et al., 2011). Chi et al. reported that MPO-positive cells probably represented neutrophils with a high proportion (38.05%) in the peritoneum of turbot stained by potassium iodide and oxidized pyronine Y (Chi et al., 2017). Other investigators have reported that MPO is not detectable after the differentiation of monocytes into macrophages (Kumar et al., 2004). This study used high-resolution electron microscopy, which revealed that ETs were released from two types of peritoneal cells. One type of cell with many fusiform peroxisomes in the cytoplasm was suggested to be neutrophil-like. The other had blunt pseudopods on the plasma membrane and nuclei consisting of a peripheral rim and central aggregates of heterochromatin with strands of euchromatin that possessed no peroxisomes and were likely phagocytes.

In summary, we established a method that facilitated visualization and quantification of ET formation *in vivo* of fish by a small animal imaging instrument. Stimulation with antigen, Alum, and FIA led to neutrophil “swarming” around adjuvant aggregates and induced peritoneal ETs. Moreover, Alum and oil adjuvants increased the release of ETs during the early time points post-administration of vaccines. In addition, by ultrastructural observation, we suggest that neutrophils and macrophage-like cells are involved in ET formation in the peritoneal cavity.

## Data Availability Statement

The original contributions presented in the study are included in the article/supplementary material. Further inquiries can be directed to the corresponding author.

## Ethics Statement

The animal study was reviewed and approved by the Committee of the Ethics on Animal Care and Experiments at the Ocean University of China.

## Author Contributions

QL, HC, and RAD participated in the conception and design of this study. QL, QG, XS, YS, and HC performed the experimental and statistical analyses. QL and HC wrote the original draft. JX, XT, XS, WZ, and RAD reviewed and edited the manuscript. HC, JX, XT, XS, and WZ provided the funding. All authors read and approved this version of the final manuscript and acknowledge the integrity of this work.

## Funding

This research was jointly supported by grants from the National Natural Science Foundation of China (31872594 and 31730101) and the National Key Research and Development Program of China (2019YFD0900101 and 2019YFD0900102).

## Conflict of Interest

The authors declare that the research was conducted in the absence of any commercial or financial relationships that could be construed as a potential conflict of interest.

## Publisher’s Note

All claims expressed in this article are solely those of the authors and do not necessarily represent those of their affiliated organizations, or those of the publisher, the editors and the reviewers. Any product that may be evaluated in this article, or claim that may be made by its manufacturer, is not guaranteed or endorsed by the publisher.
